# Child Wellbeing during the COVID-19 Pandemic: A Multi-cohort Comparison and a Multi-informant Genetic Study

**DOI:** 10.1007/s10519-025-10223-3

**Published:** 2025-06-05

**Authors:** Hekmat Alrouh, Josjan Zijlmans, Michiel Luijten, Hedy van Oers, Jacintha M. Tieskens, Christel M. Middeldorp, Arne Popma, Tinca J. C. Polderman, Meike Bartels

**Affiliations:** 1 Department of Biological Psychology, Faculty of Behavioral and Movement Sciences, Vrije Universiteit Amsterdam, Van der Boechorststraat 7-9, Room MF-H557, Amsterdam, 1081 BT The Netherlands; 2 Amsterdam Public Health Research Institute, Amsterdam, The Netherlands; 3 Department of Public Administration and Sociology, Erasmus University Rotterdam, Rotterdam, The Netherlands; 4 Department of Child and Adolescent Psychiatry & Psychosocial Care, Amsterdam University Medical Center, Vrije Universiteit Amsterdam, Amsterdam, The Netherlands; 5 Child and Adolescent Psychiatry & Psychosocial Care, Amsterdam University Medical Center, University of Amsterdam, Emma Children’s Hospital, Amsterdam, The Netherlands; 6 Epidemiology and Data Science, Amsterdam University Medical Center, Vrije Universiteit Amsterdam, Amsterdam, The Netherlands; 7 LUMC Curium– Child and Adolescent Psychiatry, Leiden University Medical Center, Leiden, The Netherlands; 8 Department of Youth and Family, Department of Research, Arkin Institute for Mental Health, Amsterdam, The Netherlands; 9 Levvel, Academic Centre for Child and Adolescent Psychiatry, Amsterdam, The Netherlands; 10 Child Health Research Centre, University of Queensland, Brisbane, QLD Australia; 11 Child and Youth Mental Health Service, Children’s Health Queensland Hospital and Health Service, Brisbane, QLD Australia; 12 Karakter Child and Adolescent Psychiatry University Centre, Nijmegen, The Netherlands; 13 Department of Child and Adolescent Psychiatry, University Medical Center Groningen, University of Groningen, Groningen, The Netherlands

**Keywords:** Wellbeing, COVID-19, Multi-rater Twin Model

## Abstract

The impact of the COVID-19 pandemic on the wellbeing of 26,555 Dutch children and adolescents (ages 8–18, 50% female, 89% with parents born in the Netherlands) was investigated using three cohorts: a general population twin sample (NTR), a general population sample (KLIK), and a clinical sample (DREAMS). Data were collected in seven waves between 2020 and 2023. Linear mixed models were employed to examine changes in wellbeing, twin models were used to estimate genetic and environmental contributions, and a psychometric model was employed to explore potential rater bias. A 6.5% drop in wellbeing was observed at the onset of the pandemic in the NTR sample, followed by partial recovery but not a return to pre-pandemic levels. Mean wellbeing scores were consistently lower in the clinical cohort (DREAMS), which also showed different effects of age, gender, and parental educational attainment compared to the two general population samples (NTR and KLIK). Increased disagreement between fathers’ and mothers’ ratings during lockdown was also identified. Genetic factors were found to account for 26–28% of the variance in wellbeing during the pandemic, and 34–35% before and after. Shared environmental factors were higher during the lockdown period (60–62%) compared to before and after the lockdown (45–49%), indicating the key role of family and home environment in that period. Multi-rater analyses suggested that part of this increase in shared environmental variance likely reflects rater bias rather than true environmental influences. These findings highlight that children in psychiatric care may face additional challenges compared to their peers and emphasize the importance of multi-rater assessments. Results suggest that both genetic predispositions and environmental disruptions should be considered when developing strategies to support child wellbeing during crises.

**Supplementary Information:**

The online version contains supplementary material available at 10.1007/s10519-025-10223-3.

## Introduction

The COVID-19 pandemic and the associated lockdown measures, aimed at limiting its spread, have presented significant challenges for children and adolescents (hereafter referred to as children) worldwide (Fegert et al. 2020). Social relationships play a crucial role in the wellbeing of developing children (Konu et al. 2002), and the closure of schools and other restrictions severely limited opportunities for social interaction among school-aged children. Therefore, numerous empirical studies have investigated the impact of the pandemic on mental health and wellbeing of youth, yielding mixed findings. These studies highlight diverse influences, including age-related differences, individual factors like socio-economic status, family functioning, and social support, as well as national factors such as the intensity of protective measures and infection dynamics (Wolf and Schmitz 2023). The majority of studies indicate a negative effect on mental health of children in the general population, particularly concerning depression and anxiety (Barendse et al. 2022; Racine et al. 2021; Zolopa et al. 2022). However, other studies showed no significant changes in certain mental health measures such as wellbeing and emotional problems (Raw et al. 2021; Vira and Skoog 2021) and even significant improvement with regards to wellbeing (Recchi et al. 2020) and certain symptoms such as substance use and anxiety (Giménez-Dasí et al. 2021; Pelham et al. 2021). It is also relevant to distinguish between studies relying on self-reported measures versus those drawing on clinical data or diagnoses. Self-report surveys typically capture a broad range of emotional and behavioral difficulties, including mild or transient symptoms that may never reach clinical thresholds. By contrast, clinical data generally reflect more severe or functionally impairing conditions, necessitating formal diagnoses or hospital visits. This discrepancy in data sources can contribute to the mixed findings in the literature regarding the scope and severity of children’s mental health challenges during the pandemic (Penninx et al. 2022; Sun et al. 2023). Children with pre-existing mental health problems were particularly vulnerable with increased internalizing symptoms during the pandemic compared to the general population (Fischer et al. 2022). Additionally, youth in psychiatric care have experienced enduring negative effects on their mental health in the later stages of the pandemic compared to their peers in the general population (Zijlmans et al. 2023). Increased demand for child and youth mental health services has also been reported (Tedja et al. 2023). Given the reciprocal relationship between mental health and wellbeing (Bartels et al. 2013; Baselmans et al. 2019; Okbay et al. 2016), it is essential to explore how these trends impact the wellbeing of youth, both in the general population and among those with pre-existing mental health problems. Indeed, strong wellbeing can buffer individuals against developing psychiatric symptoms, while psychological distress can compromise overall wellbeing. This perspective also aligns with international policy priorities, such as the United Nations Sustainable Development Goals (SDG 3), which explicitly promote “good health and wellbeing” for all. Consequently, focusing on wellbeing outcomes provides a broader lens to inform both public health interventions and long-term recovery strategies following crises.

While some studies have reported no change or even an increase in wellbeing during the pandemic (Dabravolskaj et al. 2021; James et al. 2021), the majority indicate a significant decline (Ehrler et al. 2021; Magson et al. 2021; Mastorci et al. 2021; Thorisdottir et al. 2021). For example, a study in Australia on adolescents aged 13–16 years found a significant 10% drop in wellbeing (measured with the Student’s Life Satisfaction Scale) between the year prior to the pandemic and two months after lockdown (Magson et al. 2021). Similarly, a larger population-based study in Iceland, involving 59,701 adolescents aged 13–18, reported a 5.4% decrease in wellbeing (measured using the Short Warwick Edinburgh Mental Wellbeing Scale) between 2018 and 2020 (Thorisdottir et al. 2021). Some of these effects were persistent after the pandemic, particularly with regards to peer relationships and future life expectations (Kozák et al. 2023) However, no studies to date have examined the longitudinal stability and change in wellbeing levels throughout the various stages of the pandemic, including the period after the cessation of lockdown restrictions. It is possible that the effects observed in these studies represent acute stress reactivity, with potential resilience developing in the chronic phase (McEwen 2017), especially considering that adolescence is characterized by greater resilience compared to other life stages (Pomatto and Davies 2017). Notably, while mental health problems in children from the general population appear to have begun to normalize towards the end of the lockdown measures, elevated levels persist in those already receiving psychiatric care (Zijlmans et al. 2023).

**Fig. 1:**
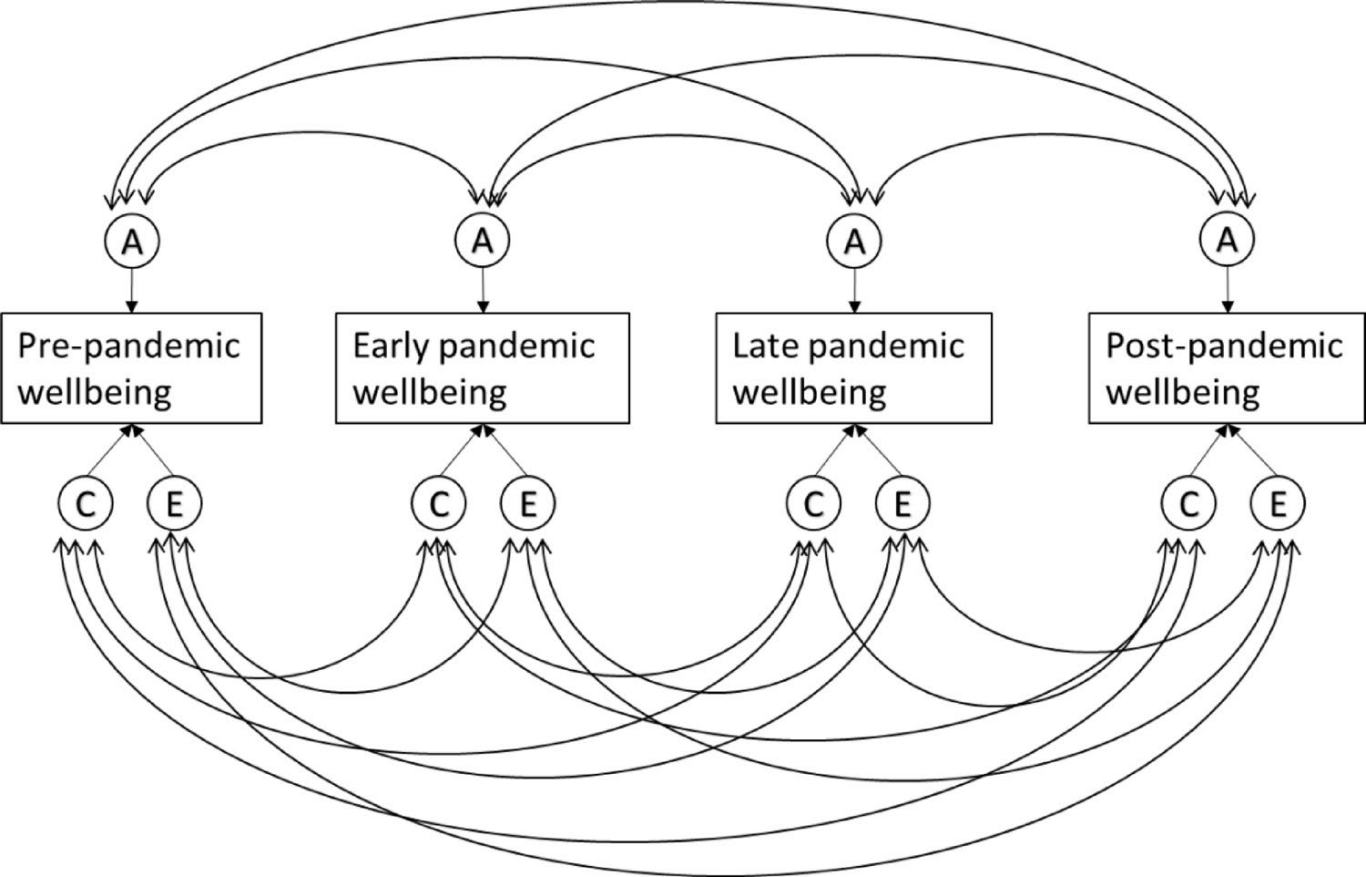
Correlated factors model

In addition to examining the changes to the mean levels of wellbeing in the population, we can view the pandemic as a natural experiment to observe possible changes in the genetic architecture of mental health and wellbeing. Previous studies have shown that differences in wellbeing are influenced by both genetic and environmental factors, which respectively explain about 30-40- and 60–70% of individual differences in wellbeing (Bartels and Boomsma 2009; Baselmans et al. 2018; van de Weijer et al. 2022b). The pandemic and its associated lockdown measures constituted a substantial and abrupt change to the environment of the entire population. Consequently, twin data collected before, during, and after the pandemic can be leveraged to provide insight into how genetic and environmental influences behave during substantial environmental change. This can be achieved by utilizing a correlated factors twin model (see Fig. 1). In this model, individual differences in a trait are decomposed into additive genetic (A) influences, shared environmental (C) influences (i.e., factors that increase similarity among siblings), and non-shared environmental (E) influences (i.e., factors that increase differences).For example, it is possible to examine the overlap in genetic factors that influence the levels of wellbeing before and during the pandemic, where less than complete overlap can indicate possible gene-environment interaction effects. Such a result would suggest that different genes express their influence on wellbeing under different environmental circumstances.

This perspective aligns with findings from other natural experiments where abrupt societal changes altered the balance between genetic and environmental influences. For example, Rimfeld et al. demonstrated that the end of the Soviet regime and the shift toward a meritocratic capitalist system in Estonia led to a significant increase in the proportion of variance in educational attainment and occupational status explained by genetic factors (Rimfeld et al. 2018). This finding illustrates how the broader social context can moderate the expression of genetic potential, reinforcing the relevance of examining how genetic and environmental influences on wellbeing may fluctuate during substantial environmental disruptions such as the COVID-19 pandemic.

**Fig. 2:**
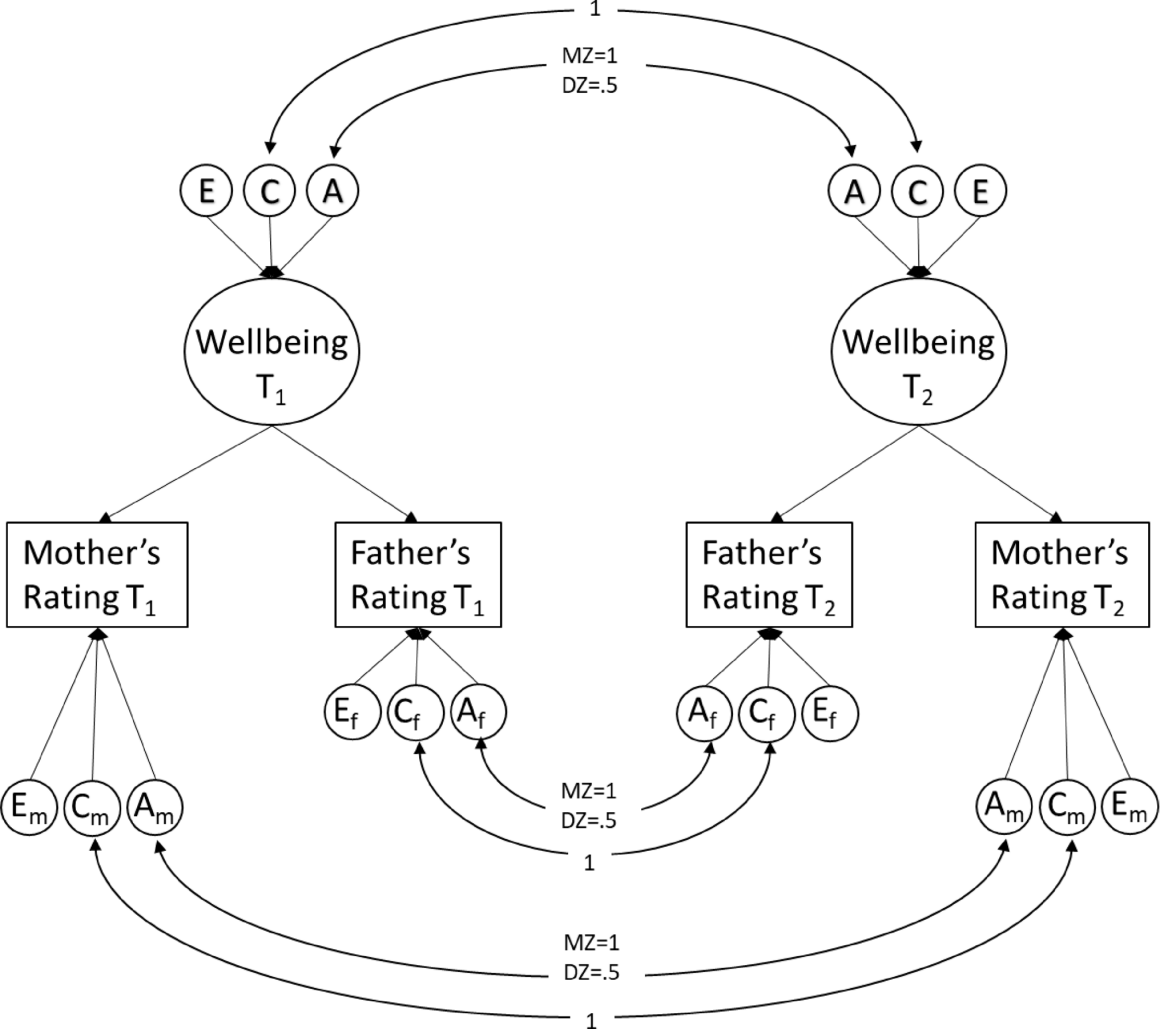
Multi-rater psychometric model using ratings from fathers and mothers

Studies on optimism and meaning in life in adults that employed this type of design showed a slight decrease to the levels of genetic variance and a slight increase in environmental variance at the start of the pandemic, with the small changes resulting in an overall stable estimate of heritability (i.e., the relative proportion of individual differences explained by genetic factors) (de Vries et al. 2022). This study however reported lower than unity genetic correlations across time (0.75 for optimism and 0.63 for meaning in life) suggesting gene-environment interactions, where the expression of genes that influence optimism and meaning in life differs before and during the pandemic. A similarly designed study on quality of life (QoL) that involved adult twins in addition to their family members showed a decrease in genetic variance and an increase in environmental variance, resulting in a decrease in heritability during the pandemic. In addition a genetic correlation of 0.58 was reported between pre-pandemic and pandemic QoL scores, indicating different genes involved in QoL before and during the initial phase of the pandemic (van de Weijer et al. 2022a). These studies were conducted on adult self-report data and covered only the early stages of the pandemic. The question remains as to how these changes would look when examining them in the youth population, and how they evolve through the various stages of the pandemic. To answer this question, we will utilize single rater longitudinal twin modeling to estimate the longitudinal genetic architecture of wellbeing through a correlated factor model (see Fig. 1). In addition, to gain insight into rater agreement and genetic and (shared) environmental effects, we used a psychometric multi-rater twin model (Bartels et al. 2004). Unlike conventional single-rater twin models, the multi-rater psychometric model (see Fig. 2) explicitly separates the variance in child behavior ratings into two distinct components: common variance (representing agreement between raters) and unique variance (representing disagreement between raters). The common variance is conceptualized as the latent, true child phenotype observable to both parents (rater agreement), and is decomposed into additive genetic (A), shared environment (C), and non-shared environment (E) components. The residual (disagreement) variance captures the unique perceptions or biases of each rater and is further decomposed into rater-specific ACE components. The rater-specific additive genetic component (A_m_ and A_f_) reflects genuine child behaviors that are expressed uniquely in the presence of one parent due to specific child-parent interactions or contexts not equally accessible to both parents. Conversely, rater-specific shared environmental component (C_m_ and C_f_) reflect systematic biases or consistent differences in parental rating standards and perceptions, such as one parent consistently rating behaviors higher or lower than the other parent due to their expectations, standards, or beliefs about child behavior. Finally, rater-specific non-shared environmental effects (E_m_ and E_f_) capture measurement error or truly idiosyncratic experiences and interactions that occur uniquely between one parent and the child. By leveraging the genetically informative nature of twin data, this psychometric approach enhances interpretability by differentiating true shared environmental influences on child wellbeing from rater-specific biases and errors, thus providing more precise estimates of how parental perceptions of child wellbeing vary throughout the pandemic. See Bartels et al. for a more detailed and elaborate explanation of the multi-rater model (Bartels et al. 2007).

The current study combines data from three cohorts in the Netherlands: a cohort of youth receiving psychiatric care, a general population cohort, and a general population twin sample. In this study, we opt to quantify wellbeing as (parent reported) QoL, given its significant phenotypic and genetic correlations with other aspects of wellbeing (Bartels and Boomsma 2009; Baselmans et al. 2019; Bjørndal et al. 2023). By utilizing measurements obtained before the pandemic and at five different time periods during the pandemic, we aim to examine the changes in wellbeing levels, quantified as QoL, among children during the COVID-19 pandemic, as well as the role of age, gender, and socioeconomic status, indexed by parental educational attainment (PEA), as factors affecting those levels. Furthermore, by incorporating genetically informed data in a longitudinal design, we aim to assess the role of genetic and environmental factors in the stability of wellbeing throughout the pandemic.

## Methods

This study was pre-registered on the Open Science Framework (OSF) prior to data access. Pre-registration details, including objectives, methodology, and analysis plan, are available at [https://osf.io/vskwh].

### Sample

The Dutch consortium Child and Adolescent Mental Health and Wellbeing in times of the COVID-19 pandemic (CAMHWB-19) was established to examine the impact of the COVID-19 pandemic on mental health and wellbeing of children in the Netherlands. Previous reports have focused on mental health outcomes during the first two years of the pandemic (Fischer et al. 2022; Zijlmans et al. 2023). The study sample comprises three cohorts within the consortium: (1) The Netherlands Twin Register (NTR), (2) the Quality of Life in Clinical Practice group (KLIK), and (3) the Dutch Research in Child and Adolescent Mental Health (DREAMS) consortium.

The NTR is an extensive database established in 1987 to facilitate research on the causes of individual differences in human traits and health outcomes. It contains a large collection of data from twins and their families, covering various disciplines such as genetics, psychology, epidemiology, and biomedical research. Invitations to complete an online survey for each wave of the COVID survey were sent to both parents of twins aged 7, 9, and 12. Reminder emails were sent to non-respondents within a month of the initial invitation, and each wave closed within three months of its initiation. Twins in the NTR cohort with a handicap that interfered with normal daily function, along with their twin siblings (*n* = 1,054; 2.3% of the sample), were excluded. For the baseline measure of wellbeing, we included measurements from 2008 onwards obtained from longitudinal data collected in the NTR. For individuals with multiple pre-pandemic measurements, we selected the latest pre-pandemic measurement to minimize the time gap between baseline and pandemic measures for that individual. Supplementary Table S1 display baseline wellbeing levels by the year of measurement for the NTR cohort.

KLIK consists of a representative sample of the Dutch pediatric population, created with the goal of validating Dutch pediatric survey instruments. Children and their parents were invited to participate through an independent online survey panel called PanelInzicht (Luijten et al. 2021), and data were collected through the research website of the KLIK portal (www.corona.hetklikt.nu). A two-step random stratified sampling method was used to ensure that the child samples were representative (within 2.5% of the Dutch population) on key demographics; sex, age, ethnicity, social class, and educational level (the latter only for adolescents).

DREAMS is a collaboration between four academic child psychiatric centers in the Netherlands (Amsterdam, Groningen, Leiden, Nijmegen) together covering the northern, western, and eastern part of the Netherlands (Zijlmans et al. 2023). Children receiving psychiatric care and their parents were invited to participate by e-mail through their psychiatric center. Reminder emails were sent to non-responders two weeks after the initial invitation.

It is important to note that, unlike the NTR cohort which is longitudinal, both the KLIK and DREAMS cohorts are cross-sectional in nature. That is, each wave consists of a newly sampled group of participants with minimal overlap across waves. Consequently, individual-level longitudinal analyses, such as variance decomposition over time, are only feasible within the NTR cohort. This design distinction is relevant for interpreting comparisons across cohorts and should be kept in mind when assessing temporal trends in KLIK and DREAMS data.

#### COVID-19 Pandemic Related Data Collection

**Fig. 3:**
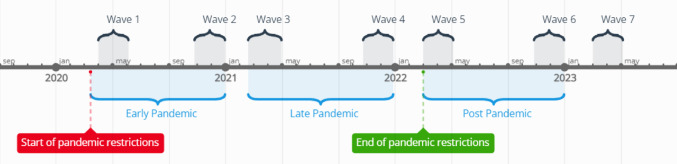
Timeline of surveys and major COVID-19 restrictions in the Netherlands

Data were collected for all three cohorts in seven waves, approximately once every six months. The NTR cohort did not participate in the seventh wave, while KLIK and DREAMS cohorts did not participate in the first wave, resulting in a total of six measurements for each cohort. The data collection periods were as follows: (1) April-May 2020, (2) November-December 2020, (3) March-April 2021, (4) November-December 2021, (5) March-April 2022, (6) November-December 2022, and (7) March-April 2023 (see Fig. 3). In each wave, parents of children meeting the inclusion criteria for the three cohorts received an email with the survey. Measurement 1 took place during the first peak of the pandemic when the initial lockdown was imposed in the Netherlands, resulting in the closure of all schools. Measurement 2 occurred during a partial lockdown with primary schools reopened. Measurement 3 was conducted during another partial lockdown with the addition of a nighttime curfew. Measurement 4 was again during a (partial) lockdown, with schools still open but followed by their closure on December 14th, immediately after data collection. Measurement 5 took place after the end of all pandemic restrictions when schools were open, people were allowed to work from the office, and face masks were no longer mandatory in most public spaces. Measurements 6 and 7 occurred well after the end of the pandemic.

### Measures

Wellbeing: Parental reports were used to assess wellbeing using a Dutch version of Cantril’s Self-Anchoring Striving Scale (Cantril 1965). This instrument asks respondents to indicate on a zero to ten Likert scale where their child’s life fell: “The 10 means the best life-, and the 0 means the worst life you can imagine.” The first survey wave for the KLIK and DREAMS cohorts did not include the Cantril Ladder scale. In the NTR cohort, mother’s report was used when both parental reports were available, whereas in the KLIK and DREAMS cohorts, only one parental report was available. For NTR, raters were 43% mother only, 6% father only, and 51% both parents. For KLIK, raters were 63% mother, 36% father, and 1% other. For DREAMS, raters were 85% mother, 13% father, and 2% other.l,

PEA: The highest educational attainment achieved by either parents was used as a proxy for socioeconomic status (Broer et al. 2019; Cowan et al. 2012), and was measured by asking participants: “What is the highest educational level that you have completed?” The education level was coded as three categories: (1) Low (elementary education, several years of lower general secondary school, lower vocational training, graduated from lower general secondary school, or lower vocational training), (2) Middle (graduated from upper general secondary school or completed intermediate vocational education), and (3) High (completed higher vocational education or university degree). Education attainment was obtained separately for each parent and transformed into a combined PEA variable based on the higher educational attainment between the two parents.

### Statistical Analysis

Pandemic Effect on Mean Levels.

A linear mixed model (LMM) analysis was performed for each cohort separately using the nlme package (version 3.1.164) in R (version 4.3.3). The model was fit using maximum likelihood estimation. The following equation was used to describe the model:

Y = (β1 + b1_i_ ) + β2 * wave + β3 * sex + β4 * age + β5 * PEA + β6 * rater + e_i_.

where Y is the wellbeing score, wave is the survey wave number and is equal to zero for baseline measurement, PEA is parental educational attainment, e_ij_ is the error term, β1,…, β6 are the fixed effects (population averages), and b1_i_ is the random intercept for individuals. In the NTR cohort, these individuals are twins with clustered observations (same family, same rater). Hence the random intercept is nested within family to capture random effects on both the individual and twin pair levels.

### Longitudinal Twin Models

In the NTR cohort, a longitudinal genetically informed analysis was conducted using measurements obtained before, during, and after the pandemic to investigate the impact of the pandemic on the genetic architecture of wellbeing. Twin models are based on the difference in genetic similarity between monozygotic (MZ) and dizygotic (DZ) twin pairs. MZ twins share virtually all their genes, while DZ twins share half on average. This difference allows for the decomposition of phenotypic variance and covariance (e.g. between time points) into genetic and environmental components. The additive genetic variance (A) represents the variance attributable to independent effects of alleles on the phenotype, while non-additive genetic variance (D) reflects allele interactions at the same or different loci. Environmental variance includes shared environmental influences (C) and nonshared environmental influences, which are comprised of unique environmental factors and measurement error (E). An ACE or ADE model choice was made based on the ratio of twin correlations, where an ACE model was selected if the MZ correlations were lower than twice the DZ correlations, indicating the presence of common environmental effects. Conversely, an ADE model was used if the MZ correlations were higher than twice the DZ correlations, suggesting dominant genetic effects (Neale and Cardon 2013). The Correlated Factors Model (Fig. 1), an extension of the classical univariate twin model, was employed to examine the underlying factors contributing to stability in wellbeing throughout the pandemic. This multivariate approach allows for the decomposition of covariance between the trait of interest at multiple time points into genetic, shared environmental, and nonshared environmental components. For the correlated factors model, we used ratings from one parent, utilizing maternal ratings for children with ratings from both parents. Cross-twin cross-trait correlations obtained from MZ and DZ twin pairs were used to inform the model to estimate the genetic and environmental influences on the phenotypic relationship between wellbeing over time. The twin models were fitted using the OpenMx package (version 3.9.9) in R (version 4.3.3).

### Multi-rater (Psychometric) Model

Assessments of psychopathology and wellbeing in children often rely on reports from either mothers or fathers. Parents are of course a valuable source for information about their children, but as external raters, they will probably individually provide an incomplete picture of the phenotype of interest, and at worst, introduce rater bias (Bartels et al. 2004a, b). This bias, reflecting the rater’s characteristics and tendencies to over- or underestimate certain behaviors, will add variance to the child’s phenotype (rater variance). A unique feature of twin studies is that, in the case that parents rate both children of a twin pair, this bias (showing up as shared environmental effects) can be identified and quantified when data of multiple raters are combined. In addition, each parent may provide an additional unique perspective on the child’s phenotypes, stemming from either observing the child in a different environment than the other parent, or by having different interactions with the child (Achenbach et al. 1987). In this case, the parental unique perspective will contribute to a rater specific additive genetic variance (Bartels et al. 2004a, b). In studies involving multiple raters, such as both parents rating both twins, unbiased estimates of shared environmental factors can be obtained by assessing the level of agreement between parents. Previous multiple-rater studies have suggested that rater bias may account for approximately 10–30% of the variance in phenotypic ratings (Abdellaoui et al. 2008; Bartels et al. 2007; Boomsma et al. 2005; Hudziak et al. 2003; van der Valk et al. 2003; van Grootheest et al. 2007). However, these studies have primarily focused on broad measures of internalizing and externalizing problems or specific domains of psychopathology, and estimates for wellbeing and quality of life measures are lacking. Given the subjective nature of wellbeing assessment, it is important to examine this phenotype through the lens of multiple raters to better understand the impact of rater bias. Furthermore, as we expect an increase in shared environment components of wellbeing for children during the pandemic as both twins spend more time in the same home environment, we aim to distinguish between real shared environmental effects (reflected in the C component of agreement portion in the multi-rater model) and that of rater bias (reflected in the C component of the disagreement portion).

A 4 × 4 variance-covariance matrix was estimated for each of the four periods and two zygosity groups (monozygotic and dizygotic) while including sex and age as covariates. The matrices contained covariance between twin 1 and twin 2 as rated by each parent (same-twin same-rater), covariance between parental ratings for each twin (same-twin cross-rater), and cross-covariance between parents’ ratings of different twins (cross-twin cross-rater; e.g., father rating of twin 1 and mother rating of twin 2). The covariances were constrained in two ways: (1) ensuring that parental rating covariances for each twin were equal across zygosities and (2) ensuring that cross-twin cross-rater covariances were equal across twin order. The most parsimonious models (with more degrees of freedom) that satisfied these constraints were selected for subsequent genetic analyses. To separate common and specific aspects of parental ratings on the child’s behavior, a psychometric genetic model was employed based on previous studies by Hewitt et al. (1992) and Bartels et al. (2007) (see Fig. 2). This model assumed a common component in the phenotype assessed by both parents (rater agreement) and a unique component reflecting each parent’s individual assessment of the child’s phenotype (rater disagreement). The total variance of each parent’s ratings was decomposed into common (agreement) and rater specific parts. The common component was further decomposed into additive genetic, shared environmental, and nonshared environmental components. The common additive genetic variance represented the heritability of the trait assessed by both parents. The specific component of each parent’s ratings was also decomposed in the same manner, with the additive genetic variance representing the heritable behavior of the child expressed uniquely in the presence of each parent. Rater bias was reflected in the proportion of shared environmental variance specific to each rater.

To analyze the genetic architecture and rater-specific perspectives, we collapsed survey waves into four periods (pre-pandemic, early pandemic, late pandemic, post-pandemic) to improve statistical power and model estimation. Table S2 shows number of twin pairs and available raters in each period in the genetic architecture analysis. To assess changes in rater effects before, during, and after the pandemic, we conducted a post hoc linear mixed models analysis of rater effects on the mean by period.

The statistical analyses conducted for this study were primarily confirmatory. The confirmatory analyses included the genetic architecture and multi-rater psychometric models, which were designed to test predefined hypotheses regarding the influences of genetic and environmental factors on child wellbeing across different pandemic periods. However, the post-hoc analysis of the rater effect by period was exploratory in nature. This post-hoc analysis aimed to assess changes in rater effects on the mean wellbeing scores before, during, and after the pandemic, which were not part of the initial hypotheses.

## Results

### Descriptive Statistics

Table 1 displays the sample characteristics for each cohort and survey wave.

**Table 1:** Sample descriptives

			Survey wave						
NTR		Baseline	1	2	3	4	5	6	7
n (total = 20,884)		17,307	3581	1173	1476	1794	1833	1662	–
Males %		50.1%	48.6%	51.3%	50.6%	48.2%	49.8%	49.2%	–
Mean age (SD)		11.2(1.8)	10.5(1.8)	10.5(2.0)	10.5(2.2)	11.2(2.1)	11.4(1.9)	11.9(2.2)	–
Parental Education	Low	11.1%	3.6%	2.5%	3.0%	2.0%	2.6%	0.9%	–
	Middle	37.2%	29.9%	33.0%	30.3%	24.0%	21.1%	29.6%	–
	High	51.6%	66.5%	64.5%	66.7%	74.0%	76.3%	69.5%	–
Rater	Mother	94.8%	86.0%	89.3%	87.3%	84.1%	82.4%	80.3%	–
	Father	60.4%	29.7%	28.7%	27.8%	28.3%	26.4%	29.1%	–

DREAMS									
n (total = 2,557)		–	–	693	495	448	312	313	296
males %		–	–	57.1%	58.4%	54.9%	52.6%	46.6%	50.3%
mean age (SD)		–	–	13.2(3.0)	13(2.9)	13(3.0)	13.3(2.9)	13.4(2.9)	13.6(3.0)
Parental Education	Low	–	–	5.1%	6.3%	6.8%	6.8%	6.5%	3.8%
	Middle	–	–	46.4%	43.4%	42.2%	38.8%	35.9%	36.5%
	High	–	–	48.5%	50.3%	50.9%	54.4%	57.5%	59.7%
Rater	Mother	–	–	86.3%	85.5%	85.7%	86.8%	83.7%	84.8%
	Father	–	–	10.8%	11.5%	12.9%	12.2%	13.0%	13.8%
	Other	–	–	3.0%	2.9%	1.4%	0.9%	3.3%	1.3%

KLIK									
n (total = 3,144)		–	–	591	445	402	505	538	663
Males %		–	–	52.0%	53.5%	52.2%	49.7%	48.5%	51.9%
Mean age (SD)		–	–	13.8(3.2)	13.6(3.3)	13.7(3.1)	13.5(3.1)	13.6(3.3)	13.5(3.1)
Parental Education	Low	–	–	9.8%	9.7%	9.5%	6.7%	7.1%	8.7%
	Middle	–	–	51.4%	45.8%	49.5%	48.6%	49.8%	47.2%
	High	–	–	38.7%	44.5%	41.0%	44.6%	43.1%	44.0%
Rater	Mother	–	–	63.3%	64.3%	65.4%	61.3%	61.5%	62.1%
	Father	–	–	35.0%	34.8%	33.1%	37.5%	37.5%	37.3%
	Other	–	–	1.7%	0.9%	1.5%	1.2%	0.9%	0.6%

Dates of covid survey waves: 1: April–May 2020, 2: November–December 2020, 3: March–April 2021, 4: November–December 2021, 5: March–April 2022, 6: November–December 2022, 7: March–April 2023.

### NTR Cohort

The NTR cohort consisted of 20,884 children—of which 17,697 had baseline measurements—with a mean age of 11.3 years. The sample was 49.6% males, and PEA levels were distributed as 11.1% low, 37.2% middle, and 51.7% high. Notably, the NTR cohort was the youngest among the three samples.

### KLIK Cohort

The KLIK cohort comprised 3,144 children with a mean age of 13.59 years and was 51.2% males. PEA levels were 8.5% low, 48.7% middle, and 42.8% high.

### DREAMS Cohort

The DREAMS cohort included 2,557 children with a mean age of 13.20 years and was 54.4% male. PEA levels were 5.9% low, 41.7% middle, and 52.4% high. As a clinical sample, the DREAMS cohort consisted of children receiving mental health services, setting it apart from the general population samples.

**Fig. 4:**
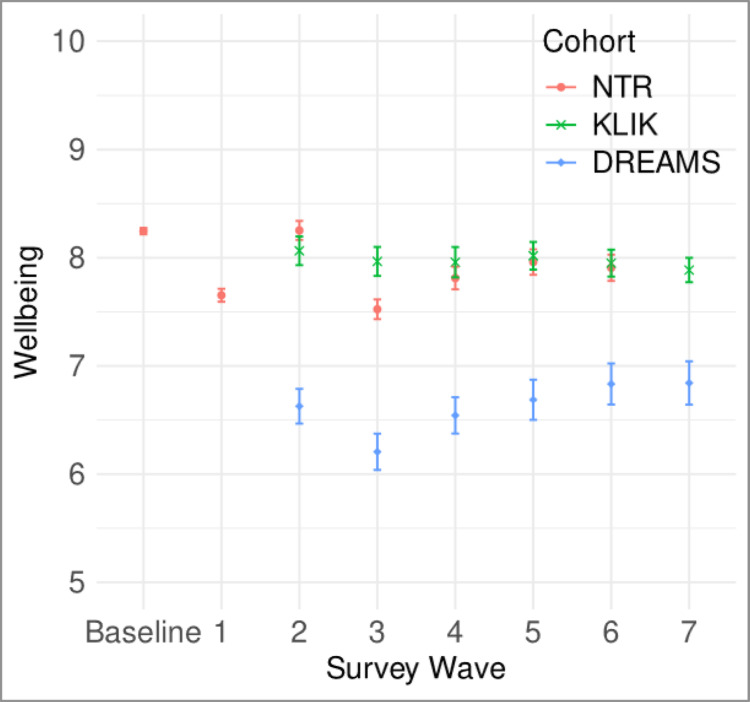
Estimated marginal means of wellbeing per survey wave for the three study cohorts

### Mean Levels of Wellbeing

Figure 4 presents the estimated marginal means for wellbeing in each cohort and survey wave obtained from LMM analysis.

### NTR Cohort

Wellbeing levels in the NTR cohort showed fluctuations across the survey waves. Compared to baseline, wellbeing was significantly lower in all waves except Wave 2. Sex effects indicated that girls had significantly higher levels of wellbeing than boys (b = 0.05, t(5017) = 3.00, *p* =.003). Age was inversely related to wellbeing, with older children reporting lower levels (b = −0.02, t(9815) = −3.69, *p* =.0002). Lower PEA was significantly associated with reduced wellbeing (low PEA: b = −0.17, t(5026) = −4.28, *p* <.0001; middle PEA: b = −0.11, t(5026) = −4.46, *p* <.0001). Fathers rated their children’s wellbeing significantly lower than mothers did (b = −0.05, t(9815) = −3.92, *p* =.0001).

### KLIK Cohort

The KLIK cohort displayed stable wellbeing levels throughout the observable period starting from October 2020. No significant sex or age effects were found. Only low PEA was significantly associated with lower wellbeing levels (b = −0.25, t(2965) = −2.71, *p* =.0067). There were no significant rater effects, potentially due to the smaller sample size and the fact that only one parent provided ratings per child.

### DREAMS Cohort

As a clinical sample, the DREAMS cohort exhibited the lowest mean levels of wellbeing among the three cohorts. Wellbeing levels fluctuated at the beginning of the observable period but stabilized towards the end. Girls reported significantly lower wellbeing than boys (b = −0.34, t(2455) = −4.99, *p* <.0001). Age was negatively associated with wellbeing (b = −0.14, t(733) = −12.03, *p* <.0001). PEA did not have a significant effect on wellbeing in this cohort. Similar to KLIK, rater effects were not statistically significant.

### Summary of Findings across Cohorts

The linear mixed model results revealed notable cohort-level consistencies and differences. Both the NTR and KLIK general population cohorts showed comparable patterns, with consistent associations between age, sex, and parental educational attainment (PEA) and wellbeing. For instance, effect sizes for age were quite small in both cohorts, though the directions of effect were opposite to each other (b = −0.021 for NTR, b = 0.007 for KLIK). Similarly, lower PEA was linked to reduced wellbeing in both general population samples (b = −0.168 in NTR, b = −0.254 in KLIK). These effects were of moderate magnitude, underscoring the importance of socioeconomic status in wellbeing outcomes.

In contrast, the DREAM clinical cohort showed distinct differences in the magnitude and direction of these associations. Notably, age had a much stronger negative effect on wellbeing in DREAM (b = −0.14), while PEA did not significantly affect wellbeing in this cohort. Additionally, the sex effect was reversed in DREAM, where girls reported lower wellbeing than boys (b = −0.344), contrary to the trend in NTR and KLIK, where girls had marginally higher wellbeing (though not significant in KLIK).

These findings highlight that, while mean levels of wellbeing were broadly similar between NTR and KLIK, DREAM consistently showed much lower wellbeing scores across all waves. These cohort-specific differences emphasize the varied impact of the pandemic on children from different backgrounds (Table 2).

**Table 2:** Linear mixed model results

		NTR (*n* = 20,884)		KLIK (*n* = 3144)		DREAMS (*n* = 2557)	
		b	p-value	b	p-value	b	p-value
Wave^a^	1	**−0.593**	**< 0.0001**	–	–	–	–
	2	0.007	0.8789	–	–	–	–
	3	**−0.722**	**< 0.0001**	**−**0.098	0.2770	**−0.421**	**< 0.0001**
	4	**−0.434**	**< 0.0001**	**−**0.106	0.2560	**−**0.085	0.3251
	5	**−0.286**	**< 0.0001**	**−**0.046	0.5982	0.059	0.5320
	6	**–0.339**	**< 0.0001**	**−**0.114	0.1884	0.205	0.0386
	7	–	–	**−**0.178	0.0317	0.215	0.0391
Sex	Female	**0.047**	**0.0027**	0.081	0.1049	**−0.344**	**< 0.0001**
Age (years)		**−0.021**	**0.0002**	0.007	0.3691	**−0.143**	**< 0.0001**
Parental Educational Attainment^b^	Low	**−0.168**	**< 0.0001**	**−0.254**	**0.0067**	0.280	0.0556
	Middle	**−0.112**	**< 0.0001**	**−**0.123	0.0190	0.039	0.5743
Rater	Father	**−0.046**	**0.0001**	0.011	0.8309	0.200	0.0398

Results in bold are statistically significant at *p* <.01.

^a^For NTR cohort, the reference period is pre-pandemic baseline, for DREAMS and KLIK cohorts, the reference wave is wave 2

^b^The reference group is High

### Changes in Genetic and Environmental Influences Associated with the Pandemic

Tables 3 and 4 present the results of the correlated factors model. Supplementary Table S3 shows the overlap of measurements across raters and pandemic periods. Given that MZ correlations were substantially smaller than twice the DZ correlations (see Table 5), an ACE model was selected to fit the data, indicating the presence of both genetic and shared environmental influences. Age and sex covariates were significant. The total phenotypic variance was significantly higher during early and late pandemic periods compared to pre- and post-pandemic. The increase in total variance during lockdown was driven mainly by the shared environment component, where the variance significantly increased from 0.52 (95% CI 0.48–0.55) at baseline to 0.86 (95% CI 0.78–0.95) and 0.80 (95% CI 0.71–0.85) during early and late pandemic respectively, and subsequently returning to baseline level at 0.50 (95% CI 0.41–0.59) post-pandemic.

**Table 3:** Multivariate phenotypic and unstandardized ACE estimates of wellbeing across 4 stages of the pandemic

		Variance/covariance				Correlation		
		Pre- pandemic	Early pandemic	Late pandemic	Post-pandemic	Pre- pandemic	Early pandemic	Late pandemic
Phenotypic	Pre-pandemic	1.06						
		(1.04–1.09)						
	Early pandemic	0.45	1.40			0.37		
		(0.44–0.5)	(1.32–1.47)			(0.32–0.4)		
	Late pandemic	0.41	0.47	1.32		0.34	0.35	
		(0.34–0.48)	(0.42–0.53)	(1.25–1.41)		(0.28–0.4)	(0.32–0.39)	
	Post-pandemic	0.49	0.49	0.57	1.1	0.45	0.4	0.47
		(0.43–0.55)	(0.44–0.55)	(0.51–0.62)	(1.04–1.17)	(0.4–0.5)	(0.36–0.44)	(0.43–0.51)
Additive genetic	Pre-pandemic	0.38						
		(0.35–0.38)						
	Early pandemic	0.21	0.39			0.55		
		(0.15–0.27)	(0.33–0.45)			(0.39–0.7)		
	Late pandemic	0.08	0.15	0.34		0.22	0.42	
		(0–0.17)	(0.09–0.21)	(0.26–0.42)		(0–0.49)	(0.25–0.46)	
	Post-pandemic	0.07	0.10	0.11	0.38	0.19	0.25	0.31
		(0–0.16)	(0.03–0.17)	(0.11–0.17)	(0.29–0.46)	(0–0.42)	(0.07–0.4)	(0.14–0.49)
Shared environment	Pre-pandemic	0.52						
		(0.48–0.55)						
	Early pandemic	0.21	0.86			0.32		
		(0.14–0.28)	(0.78–0.95)			(0.22–0.42)		
	Late pandemic	0.33	0.28	0.80		0.52	0.34	
		(0.24–0.43)	(0.23–0.35)	(0.71–0.85)		(0.37–0.67)	(0.3–0.4)	
	Post-pandemic	0.35	0.36	0.40	0.50	0.69	0.55	0.63
		(0.26–0.44)	(0.28–0.43)	(0.35–0.44)	(0.41–0.59)	(0.53–0.85)	(0.44–0.66)	(0.56–0.72)
Nonshared environment	Pre-pandemic	0.17						
		(0.16–0.18)						
	Early pandemic	0.02	0.15			0.14		
		(0.01–0.04)	(0.13–0.16)			(0.04–0.25)		
	Late pandemic	0	0.04	0.18		-0.01	0.21	
		(−0.02–0.03)	(0.01–0.06)	(0.17–0.18)		(-0.17–0.02)	(0.09–0.33)	
	Post-pandemic	0.07	0.04	0.06	0.23	0.34	0.19	0.28
		(0.04–0.09)	(0.02–0.06)	(0.04–0.08)	(0.2–0.26)	(0.2–0.46)	(0.09–0.29)	(0.18–0.37)

In terms of standardized variance components (Table 4), proportion of variance explained by genetic factors decreased during the pandemic, and returned to baseline levels in the post-pandemic period. This was accompanied by an increase in the proportion of variance explained by shared environment factors during the pandemic, similarly returning to baseline levels in the post-pandemic period. Proportion of variance explained by nonshared environmental factors slightly decreased in during the pandemic, and was higher in the post-pandemic period compared to pre-pandemic levels.

**Table 4:** Multivariate standardized ACE variance (diagonal) and covariance (off diagonal) estimates (with 95% confidence intervals) for wellbeing across 4 stages of the pandemic

		Standardized variance/covariance			
		Pre-pandemic	Early pandemic	Late pandemic	Post-pandemic
A	Pre-pandemic	0.35			
		(0.33–0.38)			
	Early pandemic	0.47	0.28		
		(0.33–0.62)	(0.24–0.32)		
	Late pandemic	0.19	0.32	0.26	
		(0–0.42)	(0.22–0.42)	(0.2–0.31)	
	Post-pandemic	0.15	0.20	0.19	0.34
		(0.14–0.18)	(0.05–0.29)	(0.09–0.28)	(0.26–0.42)
C	Pre-pandemic	0.49			
		(0.46–0.49)			
	Early pandemic	0.48	0.62		
		(0.34–0.6)	(0.57–0.66)		
	Late pandemic	0.81	0.60	0.60	
		(0.62–1)	(0.54–0.67)	(0.55–0.65)	
	Post-pandemic	0.72	0.73	0.70	0.45
		(0.56–0.87)	(0.66–0.84)	(0.64–0.79)	(0.45–0.52)
E	Pre-pandemic	0.16			
		(0.15–0.17)			
	Early pandemic	0.05	0.11		
		(0.01–0.09)	(0.09–0.12)		
	Late pandemic	0.00	0.07	0.14	
		(−0.08–0.07)	(0.03–0.12)	(0.12–0.16)	
	Post-pandemic	0.14	0.07	0.10	0.21
		(0.08–0.19)	(0.03–0.12)	(0.06–0.14)	(0.18–0.23)

Phenotypic correlation remained relatively stable throughout the various stages of the pandemic, ranging from 0.34 to 0.47 across all stages (Table 3). However, additive genetic correlations followed a diminishing trend with time. For example, the genetic correlations between the pre-pandemic period and early pandemic, late pandemic, and post-pandemic were 0.55 (95% CI 0.39–0.70), 0.22 (95% CI 0.00–0.49) and 0.19 (95% CI 0–0.42) respectively. Shared environment correlations ranged between 0.32 and 0.63, and followed an increasing trend with time. For example, to the shared environmental correlation of the pre-pandemic period was 0.32 (95% CI 0.22–0.42) with early pandemic, 0.52 (95% CI 0.37–0.67) with late pandemic, and 0.69 (95% CI 0.53–0.85) with post-pandemic period. Finally, non-shared environment correlations ranged between 0 and 0.34 but did not follow any particular trend with respect to time or period.

In terms of standardized covariance components, shared environmental influences contributed most to the stability of wellbeing throughout the pandemic, accounting for 48− 81% of the covariance in wellbeing between any two periods. Genetic influences had a relatively lower contribution to the covariance in wellbeing, ranging between 15% and 47%.

### Multi-rater Model

**Fig. 5:**
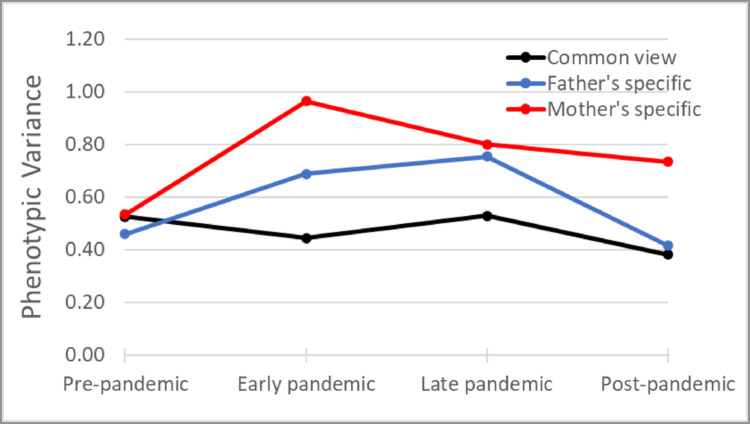
Decomposition of total phenotypic variance of wellbeing into common view and rater specific view

**Fig. 6:**
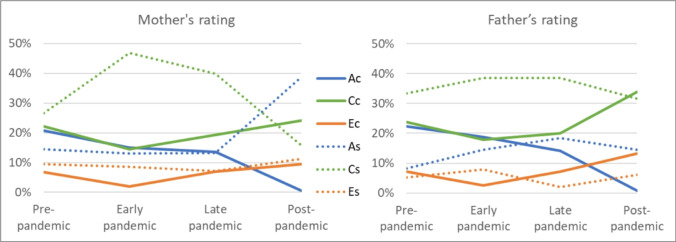
Proportion of common and rater specific ACE components of wellbeing per rater

To see whether the observed shared environmental effects in the correlated factor model is ‘true’ C or induced by rater bias, we estimated a multi-rater psychometric model for each pandemic period separately. Cross-rater correlations for the same twin decreased from 0.50 in the pre-pandemic period to 0.35 during early pandemic and gradually increased to 0.40 during late pandemic and 0.43 in the post pandemic period (see Table 5). As a result, parental common view decreased during the pandemic from 35% of total phenotypic variance at baseline down to 21% during early pandemic, then increased to 25% in late- and post-pandemic periods (Fig. 5, supplementary Table S4). Breaking down the ACE variance components (Fig. 6) reveals that the increase in specific views was accounted for by an increase in the proportion of shared environment variance specific to each parent, which is also the variance component associated with rater bias. For mothers, rater specific shared environment influences increased from 27% of the mother rated total variance at baseline to 47% during early pandemic, 40% during post pandemic period, and decreased to 16% post-pandemic. For fathers, the rater specific shared environment influences increased from 33% of father rated total variance at baseline to 39% during early and late pandemic periods, and then decreased to 36% post-pandemic. While most rater specific components returned in post-pandemic period to baseline levels, the rater specific additive genetic component– i.e. the variance component associated with a valid unique perspective—was higher in post-pandemic period compared to baseline, as it increased from 14% at baseline to 39% post-pandemic for mothers, and from 8 to 15% for fathers. In the post hoc LMM analysis of rater effects on mean wellbeing levels, fathers rated child wellbeing significantly lower than mothers in the pre-pandemic period (b = −0.06, t(9815) = −4.89, *p* <.0001). However, from the start of the pandemic onwards, father ratings were significantly higher (b = 0.15–0.26, *p* =.0001–0.0082). This pattern aligns with the higher father ratings observed in the main LMM analysis in the DREAMS cohort (b = 0.20, t(733) = 2.06, *p* =.0398), which was measured only after the start of the pandemic. Detailed results of the post hoc analysis can be found in Table S5.

**Table 5:** Twin correlations by pandemic period

		Pre-pandemic		Early pandemic		Late pandemic		Post-pandemic	
Cross rater correlation		0.50		0.35		0.40		0.43	
Cross twin		m1	f1	m1	f1	m1	f1	m1	f1
MZ	m2	0.83		0.89		0.86		0.77	
	f2	0.37	0.86	0.33	0.89	0.33	0.89	0.29	0.81
DZ	m2	0.67		0.76		0.73		0.61	
	f2	0.38	0.74	0.25	0.74	0.29	0.78	0.28	0.73

*MZ* monozygotic twins, *DZ* dizygotic twins, *m1* mother rating of twin1, *f2* father rating of twin 2

## Discussion

### Cohort Demographics

The children in the NTR (population-based twin) cohort were, on average, two years younger than those in the KLIK (population representative) and DREAMS (clinical) cohorts. The DREAMS cohort had a slightly higher percentage of males compared to the NTR and KLIK cohorts. Parental educational attainment (EA) was generally higher in the DREAMS and NTR cohorts compared to the KLIK cohort. Additionally, within each cohort, parental EA tended to increase in subsequent survey waves compared to earlier ones. This was particularly true for NTR when comparing baseline with pandemic measurements. This trend aligns with the general pattern of higher response rates among higher SES groups compared to lower ones (Agerholm et al. 2016; Demarest et al. 2013). Given that research shows that the pandemic has disproportionally impacted children with lower SES (Kozák et al. 2023; von Soest et al. 2022), it is possible that this sampling bias underestimated the impact of the pandemic on our results.

### Mean Levels of Wellbeing

This study investigated changes to mean levels of wellbeing in children throughout the pandemic, focusing on how age, gender, and parental educational attainment affect wellbeing trajectories. We also examined changes to genetic architecture of wellbeing during this period and explored parental (dis)agreement and rater bias in the parental ratings.

The two population-based samples (NTR and KLIK) demonstrated generally similar wellbeing levels, reinforcing the generalizability of our findings to the Dutch population, as the cohorts were independently sampled. Within the NTR sample, we observed a small but significant decrease in wellbeing levels at the outset of the pandemic, with some rebound after the restrictions were lifted. Wellbeing did not fully return to pre-pandemic levels, echoing the partial recovery trend noted in other studies (Ravens-Sieberer et al. 2022; Wolf and Schmitz 2023). Notably, the early pandemic waves in NTR were marked by considerable variability, driven in part by higher-than-expected wellbeing in wave 2, which may reflect the reopening of primary schools. Another potential contributor was the use of a slightly different survey format in wave 2, with omission of certain questions about negative pandemic impacts on the household, which could have influenced parent responses to the subsequent question on child’s wellbeing. By comparison, the KLIK sample showed relatively stable wellbeing scores from October 2020 onward, with no discernible drop or rebound.

It is also worth mentioning that children in the NTR sample were on average two years younger than the KLIK sample. In the Netherlands, children typically transition to secondary school around age 12, and school closures during the pandemic were shorter for primary schools compared to secondary schools. Precise classification into primary and secondary schools in our samples is difficult without additional background information due to individual differences in school enrollment and advancement. Consequently, we cannot establish exactly how many children in the NTR sample experienced the loosening of school-related lockdown measures earlier. Nonetheless, the slightly younger average age in NTR suggests that a larger portion of this sample remained in primary school during waves 2–4, potentially reducing the extent to which those children faced stricter lockdowns relative to older adolescents.

PEA emerged as an important factor in the general population cohorts. Lower levels of PEA were consistently associated with lower wellbeing, a finding that aligns with longstanding research on the importance of socioeconomic influences in shaping mental health and wellbeing in general and during the pandemic in particular (Fong and Iarocci 2020; Myhr et al. 2021; Pickett et al. 2022). Sex differences also varied by cohort. In the NTR sample, girls reported marginally higher wellbeing than boys, while in KLIK this trend did not reach statistical significance. Lastly, rater effects were not uniform across samples. In NTR, fathers tended to rate their children’s wellbeing slightly lower than mothers, whereas KLIK and DREAMS did not show statistically significant rater differences—likely due to single-parent reporting and smaller paternal response rates.

### Clinical Sample (DREAMS)

The DREAMS cohort exhibited mean wellbeing scores roughly one point lower than the general population (NTR and KLIK) cohorts across all pandemic waves. While the overall shape of the trajectory (i.e., an initial drop followed by partial recovery) paralleled that of the general population, the starting point and absolute levels were distinctly lower. Because no baseline (pre-pandemic) measurement was available for DREAMS, we cannot fully disentangle whether the pandemic itself amplified this gap; however, given prior work showing that children receiving psychiatric care generally have lower wellbeing (Dey et al. 2012), it is likely that both pre-existing vulnerability and pandemic-related stressors contributed to these differences (Hu and Qian 2021; Shoshani and Kor 2022; Wright et al. 2021).

Unexpectedly, we did not detect a significant effect of parental educational attainment (PEA) in the DREAMS sample, in contrast to the robust PEA gradient observed in the NTR and KLIK cohorts. One possibility is that children already engaged in specialized psychiatric services experience wellbeing levels that are heavily driven by clinical severity, overshadowing typical socioeconomic gradients. Another possibility is that the smaller clinical sample may have lacked power to detect an SES gradient. It is also conceivable that once a child is in clinical care, families may have been supported by structured treatment programs that partially buffer socioeconomic disadvantages—although this hypothesis warrants direct investigation. Furthermore, girls reported significantly lower wellbeing than boys, which was opposite to the observed trend in the general sample. This reversal could be linked to differences in psychopathology prevalence or severity across genders in clinical samples (Zijlmans et al. 2023). Additionally, age effects were larger in the DREAMS cohort (i.e., older children reported distinctly lower wellbeing), suggesting that adolescence with psychiatric conditions may amplify pandemic-related stressors compared to younger children.

In a longitudinal study of outpatient youth care in the Netherlands, children treated entirely during the COVID-19 pandemic exhibited higher internalizing and externalizing problem levels at both the start and end of treatment, relative to children who were treated before the pandemic. Furthermore, fewer children treated during the pandemic were classified as “recovered” from externalizing problems, despite showing similar overall treatment-related improvements in problem scores over time (Broek et al. 2025). These findings illuminate how the pandemic did not necessarily reduce the effectiveness of youth mental health treatments—i.e., the slope of improvement in problem severity appeared intact—but it did coincide with higher baseline (and ultimately end-of-treatment) levels of problems in young people already seeking care. When contextualized with our DREAMS results, this pattern supports the notion that children with pre-existing vulnerabilities are at risk of both entering the pandemic in worse overall mental health and finding it more challenging to reach subclinical levels of symptoms or fully “recover.” This aligns with broader evidence that disruptions in face-to-face therapies, rapid transitions to telehealth, and pandemic-related stressors (e.g., family distress, loss of structure, social isolation) may exacerbate ongoing psychiatric conditions. Although we did not directly collect data on the continuity of in-person vs. telehealth services, other Dutch youth-care studies underscore that increased logistical burdens on clinicians and families, pandemic-related work stress among care providers, and altered treatment formats could raise the threshold for recovery in outpatient populations (Bierbooms et al. 2020; Feijt et al. 2020).

Collectively, these results show that clinical samples are not simply a more severe version of the general population samples, as children with mental health challenges may experience social disruptions differently from their peers in the general population. Policies and interventions aiming to mitigate the pandemic’s impact on youth should be carefully tailored, recognizing that clinical samples may not display the same patterns of sex differences, socioeconomic gradients, or lockdown-related changes as broadly representative samples.

### Changes in Genetic and Environmental Influences Associated with the Pandemic

During early and late pandemic periods, shared environment factors became the majority component of wellbeing variance, ranging between 59% and 62% in those periods. The standardized covariance estimates also show that shared environment factors contributed the most to the stability of wellbeing throughout the study period. This surge in shared environment influence was expected due to twins being largely confined at home during lockdowns. In contrast, genetic factors exhibited an inverse pattern; their contribution to wellbeing variance was lowest during lockdowns, though not significantly so. Moreover, the impact of genetics on wellbeing stability across different times lessened, suggesting that wellbeing stability is less attributable to genetics during the study period. Thus shared environmental factors, heightened during the pandemic, played a more crucial role in wellbeing stability than genetic factors.

Genetic factors accounted for a significant proportion of the variance in wellbeing, and the heritability estimates (25–38%) aligned with results from earlier studies, which ranged from 22 to 47% (Bartels 2015; Baselmans et al. 2018). Furthermore, genetic correlations provided a measure of overlap in the genetic factors underlying wellbeing between any two given periods (de Vries et al. 2021), and a genetic correlation of less than 1.0 between a similar phenotype assessed at two time points is an indication of differences in underlying gene-sets influencing that phenotype. Therefore, genetic correlations that are significantly less than unity are an indicator of gene by environment interaction, where different genes express their influence under different (environmental) circumstances. Earlier studies conducted at the start of the pandemic detected this gene-by-environment interaction (de Vries et al. 2022). This was also the case in our study, in that different genes might be important for wellbeing under different circumstances. We, however, also observed that genetic correlations decreased the further apart any two periods were. This led to a situation where pre- and post- pandemic periods had the lowest genetic correlations. This finding is likely due to the combination of the age of participants, the incomplete overlap of participants between the different timepoints, and the long duration between each period, where there is at least three years between pre- and post-pandemic measurements. As a result, children who had measurements available in both those periods likely aged from 8 to 10 years old in the former to 11–13 years old in the latter. Even in the absence of a pandemic, genetic correlations between those two age groups are relatively low due to developmental changes that alter the underlying set of genes influencing wellbeing during this life phase (i.e. genetic innovation) (Bartels et al. 2004). For example, the genetic correlation for wellbeing are 0.65 between ages 7 and 10 years, and 0.41 between 7 and 12 years (Vries et al. 2024). As a result, while there is evidence of gene by environment interactions, other factors may have contributed to the this observed trend.

We reported on both the genetic and environmental correlations as well as proportion of covariance due to genetic and environmental factors (i.e. standardized covariance). The former denotes the amount of overlap between the genetic or environmental factors of two traits (or in this case, the same trait measured at different time points), and the latter denotes the extent to which genes account for correlation between the two traits. While there are certain situations where one measure is high and the other is low (see (de Vries et al. 2021) for such examples), our results show that shared environmental factors greatly overlap throughout the pandemic and also account for majority of the stability of wellbeing.

### Multi-rater Model

To obtain an observer bias free estimate of shared environmental influences on wellbeing before, during and after the pandemic, the study employed a psychometric model that differentiates potentially biased estimates from more robust estimates based on multiple raters (i.e., both parents). Results of this model indicated changes in the proportion of variance explained by the common (rater agreement) and rater-specific components of the ACE model. During the pandemic, parental agreement decreased, accompanied by an increase in rater-specific views. This challenges the notion that rater disagreement and rater bias partly arise from observing children in different environments, as most parents had to work from home and would likely observe their children in similar environments, particularly during periods of full lockdown and school closure. An alternative explanation for this increase in rater disagreement may stem from differences in child-parent interactions. Some evidence supports this hypothesis, as childcare tasks disproportionately fell on mothers during the pandemic, exacerbating gender inequality and reinforcing conventional gender parenting attitudes (Mize et al. 2021; Zamarro and Prados 2021). Conversely, it is also possible that in addition to the changes in the proportion of childcare duties, the context of interaction between children and their parents changed in mothers compared to fathers during the pandemic, resulting in an increase in rater disagreement. However, the increase in rater disagreement during the pandemic primarily resulted from an increase in the rater specific shared environmental variance, i.e. the variance component that includes rater bias (Bartels et al. 2004). This suggests an increase in rater bias of child wellbeing during the pandemic. Furthermore, the rater disagreement remained elevated in the post-pandemic period. Notably, the additive genetic component specific to mothers increased during the post-pandemic period compared to baseline, which points to the emergence of “real” unique perspectives on the child’s wellbeing. A potential explanation is the increased attention to and awareness of mental health and wellbeing as a result of the pandemic coverage (Penninx et al. 2022; Winkler et al. 2024), but it can also indicate unique behavior observed only by mothers. This increase in the rater-specific additive genetic component was more pronounced in mothers than fathers, supporting the narrative of a change in the proportion and/or context of childcare duties between mothers and fathers as a result of the pandemic (Augustine and Prickett 2022; Yerkes et al. 2024). The post hoc analysis revealed that, prior to the pandemic, mothers tended to rate child wellbeing slightly higher than fathers. However, from the start of the pandemic onwards, mothers’ ratings became significantly lower than those of fathers. This indicates that the changes in wellbeing components reported by each parent were associated with shifts in the overall reported level of wellbeing. Further research involving assessments of the wellbeing of parents and parenting load before, during, and after the pandemic can help further investigate the causes of this shift.

The multi-rater model provides valuable insights to explain the striking findings from the genetic architecture analysis. The study established evidence for an increase in rater bias for child wellbeing as a result of the pandemic. This bias led to inflated estimates for shared environmental variances during the lockdown stages of the pandemic. While these bias effects may have been exacerbated during the pandemic, they likely exist to some degree whenever parental ratings are used to evaluate child characteristics and states. Thus, the study’s results serve as a word of caution for relying on a single rater of child characteristics in twin studies, as the level of rater bias in such scenarios can significantly impact estimates of heritability and shared environmental effects, especially in cases where subjective opinion might play a role, such as ratings of child behavior, as opposed to more objective measures such as height and weight. In the case of wellbeing, the subjective nature of parental rating resulted in an overestimation of shared environment correlations and subsequently underestimated genetic correlations.

The study’s strengths include the use of multiple cohorts representing different segments of the child population, highlighting the differential effects of the pandemic on these populations, with clinical samples showing lower levels of wellbeing compared to population-based samples. The use of multiple raters for the twin cohort is another significant strength, providing a more accurate estimate of the genetic architecture of wellbeing and shedding light on the role of rater bias in assessing children’s wellbeing. Furthermore, the study offers insight into potential changes in parenting roles and parental interaction with children as a result of the pandemic, warranting further investigation.

However, it is essential to acknowledge some limitations of the study. Firstly, relying on a parent-reported measure of wellbeing may introduce biases and variations in interpretation. Inclusion of other raters, such as teachers or self-reported measures, especially in older children, would strengthen the validity of the results. Secondly, the absence of baseline measurements for some cohorts, especially the clinical population, makes it difficult to draw robust conclusions about changes in their wellbeing. Long-term data collection is necessary to establish a post-pandemic baseline, especially if the effects of the pandemic on wellbeing persist beyond the study’s duration. Thirdly, the NTR cohort had higher parental educational attainment in the pandemic waves compared to both baseline and the other two cohorts in the study (see Table 1), and selective participation across waves resulted in a larger proportion of higher-education families in later waves. This may limit the generalizability of our findings, as families with lower educational levels were underrepresented in later measurements. Educational attainment is widely used as a proxy for SES due to its relative stability and universality and lower non-response rates compared to other indicators (e.g., income, wealth) (Galobardes et al. 2006), and educational levels have been shown to explain most of the SES-related health variation (Miech and Hauser 2001). However, educational attainment may not fully capture the complexity of socioeconomic gradients. Consequently, our reliance on PEA may overlook certain nuances in SES-related vulnerabilities, and the actual impact of the pandemic on families with lower SES could be greater than our data suggest. In addition, due to the number of survey waves involved, the proportion of participants that completed all surveys was low, with around 40% overlap in the sample between any two surveys. Hence, some of the observed trends may be due to differences in the sample that are beyond the variables controlled for in the analysis such as age, sex, and PEA. Lastly, this study rests on the standard equal environments assumption of twin research, which posits that monozygotic and dizygotic twins experience equally similar environments. Although existing evidence generally supports this assumption, especially in population-based twin studies, its violation may affect estimates of ACE components.

The findings underscore the importance of considering population characteristics, socioeconomic factors, and genetic and environmental influences in understanding individuals’ wellbeing during challenging times. Moreover, the study highlights the value of using both parents as raters to assess child behavior and wellbeing, which not only mitigates potential bias but also provides valuable insight into parenting roles and familial environments. Continuous, proactive evaluation of child wellbeing is essential for accurately assessing changes during crises. The study’s results add to the existing literature on the psychological consequences of the pandemic, informing interventions and support systems aimed at promoting the wellbeing of children and their families during and after global crises.

## Electronic Supplementary Material

Below is the link to the electronic supplementary material.

Supplementary Material 1

## Data Availability

The data necessary to reproduce the analyses presented here are not publicly accessible, but are available from the corresponding author upon reasonable request. The analytic code necessary to reproduce the analyses presented in this paper is publicly accessible. Analyses were also pre-registered. Code is available at the following URL: https://github.com/hekmatov/covid-wellbeing. The pre-registration is available at the following URL: https://osf.io/vskwh.
