# Supplementary material for: Child Wellbeing during the COVID-19 Pandemic: A Multi-cohort Comparison and a Multi-informant Genetic Study
Source: Behav Genet. 2025 Jun 5;55(4):235–54. doi: 10.1007/s10519-025-10223-3 (PMC12325380; doi:10.1007/s10519-025-10223-3)
Supplement: Supplementary file 1 — Supplementary Material 1 [file 10519_2025_10223_MOESM1_ESM.docx]

**Supplementary files:**

Table S1: Mean and standard deviation of wellbeing levels for NTR baseline sample

| Year | 2008 | 2009 | 2010 | 2011 | 2012 | 2013 | 2014 | 2015 | 2016 | 2017 | 2018 | 2019 |
| --- | --- | --- | --- | --- | --- | --- | --- | --- | --- | --- | --- | --- |
| n | 1123 | 1325 | 1773 | 1525 | 1703 | 1061 | 1284 | 597 | 1469 | 2631 | 1654 | 1414 |
| Mean | 8.17 | 8.25 | 8.24 | 8.31 | 8.36 | 8.30 | 8.23 | 8.29 | 8.42 | 8.29 | 8.36 | 8.47 |
| Std. Deviation | 1.17 | 1.13 | 1.21 | 1.06 | 1.01 | 1.02 | 0.99 | 1.00 | 0.97 | 0.94 | 0.94 | 0.90 |

Table S2: Number of twins and available raters in each period in the genetic architecture analysis.

| Period | Description | n twin pairs | Maternal rating | Paternal rating | Both parents |
| --- | --- | --- | --- | --- | --- |
| Pre-pandemic: | Period before the start of the pandemic. | 8,839 | 8,383 | 5,428 | 4,972 |
| Early pandemic | First half of the pandemic restrictions period | 2,062 | 1,798 | 619 | 355 |
| Late pandemic | Second half of the pandemic restrictions period | 1,464 | 1,279 | 451 | 266 |
| Post-pandemic: | Period after the lifting of all pandemic restrictions | 1,537 | 1,259 | 442 | 200 |

Table S3: Overlap of measurements across raters and pandemic periods

|  | | Mother Report | | | | Father Report | | | |
| --- | --- | --- | --- | --- | --- | --- | --- | --- | --- |
|  |  | Pre-pandemic | Early pandemic | Late Pandemic | Post-pandemic | Pre-pandemic | Early Pandemic | Late Pandemic | Post-pandemic |
| Mother Report | Pre-pandemic | 8424 |  |  |  |  |  |  |  |
|  | School closure | 1099 | 1801 |  |  |  |  |  |  |
|  | School opening | 437 | 650 | 1289 |  |  |  |  |  |
|  | Post-pandemic | 569 | 668 | 601 | 1303 |  |  |  |  |
| Father Report | Pre-pandemic | 5001 | 571 | 229 | 256 | 5446 |  |  |  |
|  | School closure | 340 | 357 | 187 | 190 | 345 | 620 |  |  |
|  | School opening | 152 | 211 | 268 | 183 | 153 | 208 | 455 |  |
|  | Post-pandemic | 194 | 193 | 158 | 203 | 180 | 193 | 197 | 444 |

Table S4: Psychometric model results

|  | Additive genetic | | Shared environment | | Nonshared environment | | Total | | |
| --- | --- | --- | --- | --- | --- | --- | --- | --- | --- |
| **Pre-pandemic** | common | Specific | common | Specific | common | Specific | Common | Mother’s Specific | Father’s specific |
| Mother | 21% | 14% | 22% | 27% | 7% | 9% | 35% | 35% | 30% |
| Father | 22% | 8% | 24% | 33% | 7% | 5% |  |  |  |
| **Early pandemic** |  |  |  |  |  |  | 21% | 46% | 33% |
| Mother | 15% | 13% | 14% | 47% | 2% | 9% |  |  |  |
| Father | 19% | 15% | 18% | 39% | 3% | 8% |  |  |  |
| **Late pandemic** |  |  |  |  |  |  | 21% | 40% | 39% |
| Mother | 14% | 13% | 19% | 40% | 7% | 7% |  |  |  |
| Father | 14% | 18% | 20% | 39% | 7% | 2% |  |  |  |
| **Post-pandemic** |  |  |  |  |  |  | 26% | 47% | 27% |
| Mother | 1% | 39% | 24% | 16% | 9% | 11% |  |  |  |
| Father | 1% | 15% | 34% | 32% | 13% | 6% |  |  |  |

Table S5: Results of the post hoc linear mixed model analysis of rater effects on mean wellbeing levels by period.

|  |  | β | Std. Error | p-value |
| --- | --- | --- | --- | --- |
| Period | Early pandemic | -0.511 | 0.029 | <0.0001 |
|  | Late pandemic | -0.795 | 0.043 | <0.0001 |
|  | Post-pandemic | -0.506 | 0.053 | <0.0001 |
| Sex | Female | 0.046 | 0.016 | 0.0030 |
| Age (years) |  | -0.008 | 0.006 | 0.1357 |
| Parental Educational attainment | Low | -0.170 | 0.039 | <0.0001 |
|  | Middle | -0.112 | 0.025 | <0.0001 |
| Rater Father: Period | Pre-pandemic | -0.060 | 0.012 | <0.0001 |
|  | Early pandemic | 0.152 | 0.045 | 0.0007 |
|  | Late pandemic | 0.259 | 0.065 | 0.0001 |
|  | Post-pandemic | 0.217 | 0.082 | 0.0082 |
